# The Negative Relationship between Bilirubin Level and Diabetic Retinopathy: A Meta-Analysis

**DOI:** 10.1371/journal.pone.0161649

**Published:** 2016-08-29

**Authors:** Bo Zhu, Xiaomei Wu, Kang Ning, Feng Jiang, Lu Zhang

**Affiliations:** 1 Department of Cancer Prevention and Treatment, Cancer Hospital of China Medical University, Liaoning Cancer Hospital & Institute, Shenyang, People’s Republic of China; 2 Department of Clinical Epidemiology and Evidence Medicine, The First Hospital of China Medical University, Shenyang, People’s Republic of China; 3 Department of Occupational Health, Liaoning Disease Prevention and Control Center, Shenyang, People’s Republic of China; 4 Center of Health Management, Shenyang 242 Hospital, Shenyang, People’s Republic of China; Massachusetts Eye & Ear Infirmary, Harvard Medical School, UNITED STATES

## Abstract

**Objectives:**

Findings on the relationship between total bilirubin level (TBL) and diabetic retinopathy (DR) are inconsistent. Thus, we carried out a meta-analysis to investigate the relationship between TBL and the risk of DR.

**Methods:**

Relevant studies were selected from six databases up to 31 May 2016 using a search strategy. The relevant data were extracted from the included studies according to the inclusion and exclusion criteria, and the mean value with standard errors or odds ratio (OR) with 95% confidence intervals (CIs) were calculated. We compared TBL in patients with DR with that in patients with diabetes but without retinopathy (NDR), and analyzed the dose-response relationship between TBL and the risk of DR.

**Results:**

Twenty-four studies were selected in this meta-analysis. Twenty studies were included to calculate the pooled SMD, and the results showed that TBL in the DR group was lower than that in the NDR group (SMD: –0.52, 95% CI: –0.67, –0.38). Nine studies were included to calculate the pooled ORs, and the results showed that there was a significant negative relationship between TBL and the risk of DR (OR: 0.19, 95% CI: 0.14, 0.25). Six studies were included to investigate the dose-response relationship between TBL and the risk of DR, and we found a nonlinear relationship between TBL and the risk of DR. The results of our meta-analysis were found to be reliable using subgroup and sensitivity analyses.

**Conclusions:**

The results of our meta-analysis indicate that higher TBL may be protective against DR in subjects with diabetes, and TBL could be used as a biomarker to predict the risk of DR.

## Introduction

The incidence of type 2 diabetes mellitus (T2DM) is increasing rapidly. T2DM is a serious disease affecting quality of life and has become a health problem worldwide [1]. Diabetic retinopathy (DR) is a common microvascular complication of diabetes mellitus in people aged 36–69 years, and can lead to acquired blindness [2]. The incidence of DR in population-based studies ranges from 10 to 55%, and in clinic-based studies ranges from 11 to 65% [3]. Approximately 28.5% of blindness is attributed to DR in subjects aged 40 years and older [4].

More and more evidence has shown that oxidative stress is a critical risk factor in the pathogenesis of DR [5]. Oxidative stress is a pathologic state and reflects an imbalance between oxidation and antioxidation. Potent free radicals, such as superoxide radical, hydroxyl radical, and hydrogen peroxide radical, are commonly termed reactive oxygen species (ROS) and are regarded as extremely toxic [5]. The retina is a source of free radical production in T2DM, and the levels and types of oxidative stress products can predict the severity of retinopathy. The serum level of 8-OHdG in DR subjects is significantly increased compared to that in subjects with diabetes but without retinopathy (NDR) [6].

Bilirubin is mainly generated from heme degradation, and has strong antioxidant and anti-inflammatory effects on the microvasculature [7]. Bilirubin can covalently bind to albumin with a combination rate of more than 99%. It not only scavenges superoxide radical and peroxide radical, but also prevents oxidation modifications of low density lipoprotein and lipid oxidation. Animal studies have shown that bilirubin can accelerate wound healing by reducing the oxidant status of wounds in diabetic rats [6].

Some prospective studies have suggested that there is a negative relationship between total bilirubin level (TBL) and cardiovascular disease [8, 9]. A meta-analysis on the relationship between TBL and cardiovascular disease also confirmed this inverse relationship [10]. However, several studies have reported that there was no association between TBL and retinopathy of prematurity [11, 12]. In recent years, the number of studies on the relationship between TBL and DR has increased, but the effect of bilirubin on DR is unclear. Some studies showed that TBL in DR was higher than that in NDR [13–16], but other studies found no significant increase in patients with DR compared to those with NDR [17–19]. Therefore, we carried out a meta-analysis to analyze the effect of TBL on DR and evaluated the relationship between TBL and DR. To date, no meta-analyses on the relationship between TBL and DR have been carried out.

## Methods

### Search strategy and study selection

We selected appropriate studies from multiple databases (PubMed, Web of Science, CNKI (China National Knowledge Infrastructure), Wanfang database, CBM (China Biological Medicine Database), and VIP) up to 31 May, 2016 using a search strategy. The search terms used were as follows: “bilirubin” and (“retinopathy” or “retina” or “eye disease” (The filter: bilirubin [Text Word]) AND (retinopathy [Text Word]) OR retina [Text Word]) OR eye disease [Text Word]). In order to select as many appropriate articles as possible, we also reviewed reference lists in all relevant original research and review articles to identify additional potentially eligible studies.

DR was defined by an ophthalmologist following an eye examination. Fundus photographs taken for both eyes were analyzed and graded to confirm the presence and severity of DR. DR mainly included non-proliferative and proliferative types. Non-proliferative DR showed one or more of the following symptoms: microaneurysm, hemorrhage, exudates, or microvascular abnormalities; proliferative DR showed the generation of new vessels and fibrosis.

Eligible studies were included using the following criteria: (a) the study was observational (case-control, cross-sectional or cohort study) and investigated the relationship between TBL and DR; (b) the study reported either the incidence rate of DR in T2DM or TBL in DR and NDR subjects; (c) the study provided the mean value with standard errors or odds ratio (OR) with 95% confidence intervals (CIs), or the necessary data to calculate these parameters. In addition, if more than two studies investigated the same population, the latest or highest-quality study was selected. We contacted the author if the study did not provide sufficient data.

We excluded studies using the following criteria: (a) the study was not original research; (b) the study did not involve human subjects; (c) the study was a duplicate study or the data were duplicated; (d) the study did not report the relationship between TBL and DR; (e) the study did not contain sufficient data to calculate the mean value with standard errors or OR with 95% CIs.

The process of study selection and exclusion was carried out by two independent reviewers (Xiaomei, Wu and Bo, Zhu), and any discrepancies were resolved by discussion or consultation with a third reviewer (Kang, Ning).

### Data extraction and conversion

Two authors (Xiaomei, Wu and Bo, Zhu) independently extracted relevant information from the included studies according to the inclusion and exclusion criteria, and calculated the mean value with standard errors or OR with 95%CIs by obtaining data from the study or author. This information included the following: first author’s name, year of publication, study design, number of subjects, gender, age, blood components used for detection, and the corresponding estimate of the relationship between TBL and DR. To reduce the effects of the relevant factors on the results, we extracted and analyzed the adjusted OR to unadjusted OR. If the study had several ORs which were adjusted for different combinations of relevant factors, we extracted the OR which was adjusted for the greatest number of relevant factors. The method used to measure TBL in the included studies was mainly by automatic biochemical analyzer. As the units for TBL were often μmol/L and mg/dL, in order to carry out a statistical analysis, μmol/L was converted to mg/dL by dividing by 17.1.

We also determined whether there was a dose-response relationship between the risk of DR and TBL, therefore, the following information was also required: (a) the risk estimates and their 95%CIs for at least three exposure categories; (b) the median or mean TBL in each category. We extracted the exposure category and ORs with their 95%CIs from the studies, which contained the above information.

### Quality Assessment

The included studies in our meta-analysis were independently assessed by two authors (Xiaomei, Wu and Bo, Zhu). Seven items were selected from the Strengthening the Reporting of Observational Studies in Epidemiology (STROBE) statement [20], as follows:

1. Objectives: state specific objectives, including any prespecified hypotheses;
2. Study design: present key elements of study design early in the paper;
3. Setting: describe the setting, locations, and relevant dates, exposure, follow-up, and data collection;
4. Participants: Cohort study—Give the eligibility criteria, and the sources and methods of selection of participants. Describe methods of follow-up. Case-control study—Give the eligibility criteria, and the sources and methods of case ascertainment and control selection. Give the rationale for the choice of cases and controls. Cross-sectional study—Give the eligibility criteria, and the sources and methods of selection of participants;
5. Variables: clearly define all outcomes, exposures, give diagnostic criteria;
6. Statistical methods: Describe all statistical methods;
7. Main results: Report numbers of outcome events or summary measures and category boundaries when continuous variables were categorized. Give unadjusted estimates and, if applicable, confounder-adjusted estimates and their precision (e.g., 95% confidence interval).

The high quality studies met 7 items, the low quality studies met less than 5 items, and other studies were moderate quality.

### Statistical analysis

This meta-analysis was conducted using the Stata software package (Version 12.0; Stata Corp., College Station, TX). The standardized mean difference (SMD) and pooled odds ratios (ORs) were used to compare between DR and NDR group. The Chi-square-based Q-test was used to evaluate the heterogeneity among the individual studies. Heterogeneity was quantified based on I^2^, which ranged from 0% to 100% (I^2^ = 0% to 25%, no heterogeneity; I^2^ = 25% to 50%, moderate heterogeneity; I^2^ = 50% to 75%, large heterogeneity; I^2^ = 75% to 100%, extreme heterogeneity). When I^2^ was larger than 50%, a random effects model was used; otherwise, the fixed model was used.

We conducted the dose—response meta-analysis to calculate study-specific slopes (i.e., linear trends) and 95%CIs, which proposed by Greenland S. et al and Orsini N. et al [21, 22]. If the study reported exposure category by a range, the midpoint was calculated by averaging the lower and upper bound; if the lowest category was open-ended, the lowest boundary was considered to be zero; if the highest exposure category was open-ended, the width of the open-ended interval was taken to be the same as the adjacent interval.

If there was high heterogeneity between studies, we used subgroup analysis on year of publication, study design, number of subjects, gender, age and quality of study to find the source of heterogeneity. We used the sensitivity analysis to evaluate the robustness of the results in our meta-analysis. In the sensitivity analysis, we excluded each study in turn and obtained the pooled estimates from the remaining studies. The purpose of sensitivity analysis was to evaluate the effect of a single study on the overall pooled estimates. The possibility of publication bias was assessed using visual inspection of funnel plots and Egger's test, Egger's test was used to detect the asymmetry test for the funnel plot. A two-sided P value <0.05 in statistical process was considered significantly different.

## Results

### Literature search and study characteristics

**Fig 1:**
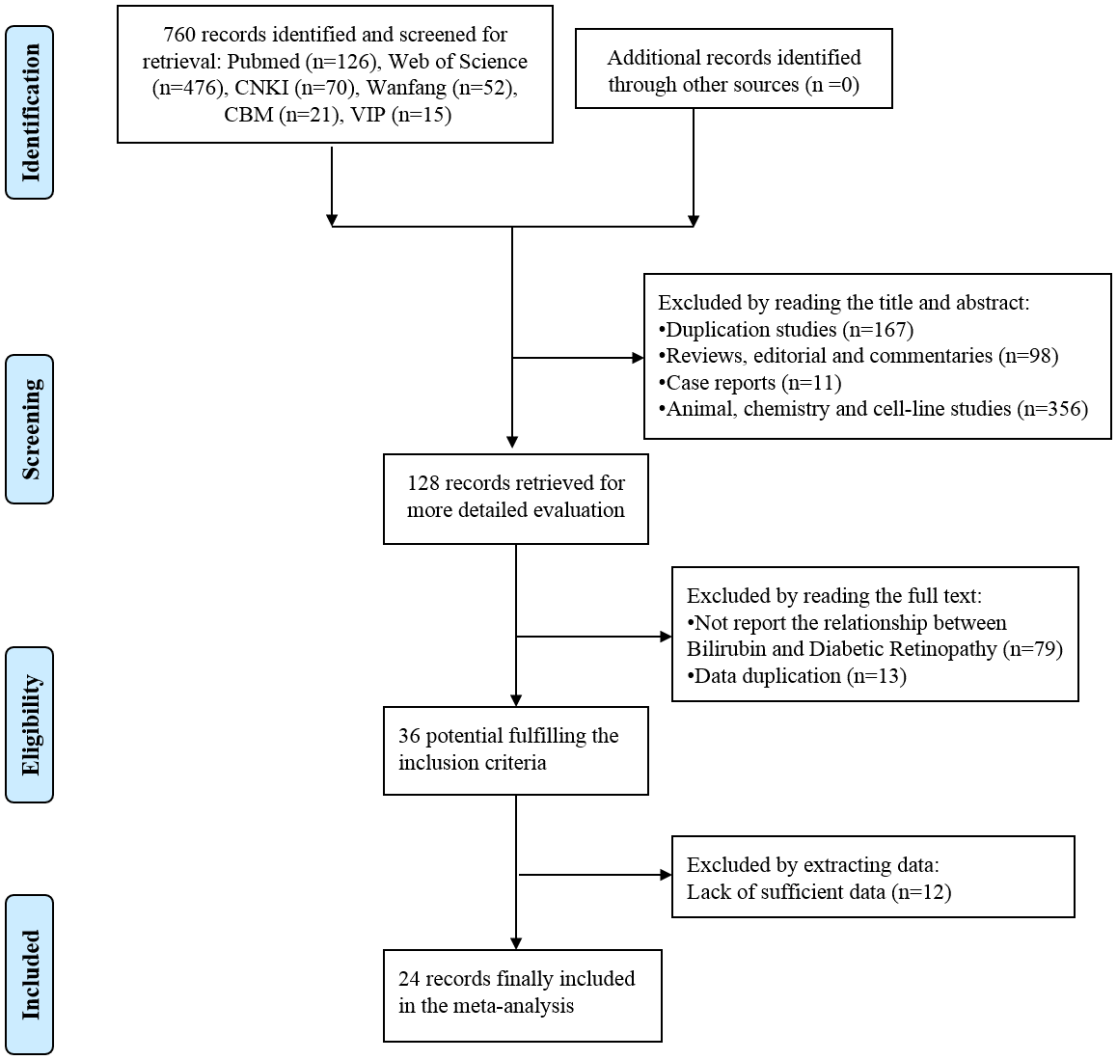
The process of study selection in our meta-analysis.

We identified 760 potential studies in six electronic databases (PubMed: 126, Web of Science: 476, CNKI: 70, Wanfang database: 52, CBM: 21, and VIP: 15) using our search strategy. We did not find any new eligible studies during a manual search of the reference lists within these studies. After reading the title and abstract, we excluded 632 studies: 167 duplicate studies, 98 reviews, editorials and commentaries, 11 case reports and 356 animal, chemistry-based and cell-line studies. After reading the full text, we excluded 92 studies, which did not report the relationship between TBL and DR or contained data from the same study population. When we extracted the data, 12 studies did not provide sufficient information. Thus, 24 studies [13–19, 23–39] were finally selected for our meta-analysis. The flow chart of the screening process is shown in Fig 1.

Of the 24 studies included, there were 16 case-control studies, 7 cross-sectional studies, and 1 cohort study. In total, 20,913 subjects were enrolled in our meta-analysis. Twenty studies included mean values and their standard errors and9 studies included ORs and their 95%CIs. Six studies provided the exposure category and ORs with their 95%CIs. The characteristics of the included studies are shown in Table 1.

**Table 1:** The characteristics of the included studies in the meta-analysis.

First Author	Year	Design	Number of subjects	Gender(Male/Female)	Age, years(mean± SD)	Blood	The criterion of DR	TBL (mean±SD, mg/dl)			Exposure categories (mg/dl)	OR (95%CI)^b^
								DR	NDR	OR (95%CI)^a^		
Dan, Zhang[23]	2015	Case-control	553	271/282	59.0 ± 11.3	Serum	Funduscopic examination	0.71 ± 0.31	0.93 ± 0.31	0.20 (0.10, 0.37)^#^	-	-
Wei, Wei[15]	2015	Case-control	100	56/44	53.9 ± 9.9	Serum	Funduscopic examination	0.88 ± 0.45	1.36 ± 0.68	0.05 (0.00, 0.81)	≤0.65	1.00
											0.66–1.23	0.78 (0.50, 1.21)
											1.24–1.59	0.69 (0.42, 1.12)
											≥1.60	0.20 (0.08, 0.51)
Fang, Chen[24]	2015	Case-control	237	134/103	58.7 ± 14.8	-	Funduscopic examination	0.59 ± 0.25	0.84 ± 0.43	0.07 (0.01, 0.33)^#^	-	-
Junwei, Cai[19]	2015	Case-control	118	49/69	54.9 ± 6.0	Serum	Funduscopic examination	0.83 ± 0.06	0.83 ± 0.06	-	-	-
Sekioka, Risa[16]	2015	Cross-sectional	674	446/228	64.7 ± 13.9	Serum	Funduscopic examination	0.65 ± 0.30	0.80 ± 0.39	0.26 (0.14, 0.49)^#^	-	-
Hamamoto, S[25]	2015	Cross-sectional	523	312/211	60.5 ± 12.3	-	Funduscopic examination	0.53 ± 0.22	0.62 ± 0.23	0.10 (0.03, 0.27)^#^	-	-
Dave, Apoorva[26]	2015	Case-control	80	46/34	53.9 ± 9.9	Serum	Funduscopic examination	0.88 ± 0.45	1.36 ± 0.68	0.05 (0.00, 0.81)^#^	≤0.65	1.00
											0.66–1.23	0.73 (0.45, 1.20)
											1.24–1.59	0.61 (0.35, 1.08)
											≥1.60	0.17 (0.06, 0.51)
Xiaojing, Shang[17]	2014	Case-control	95	52/43	58.4 ± 1.7	Serum	Funduscopic examination	0.61 ± 0.36	0.67 ± 0.30	-	-	-
Wei, Peng[27]	2014	Case-control	220	93/127	56.9	Serum	Funduscopic examination	0.50 ± 0.17	0.68 ± 0.26	-	-	-
Najam, Syeda Sadia[28]	2014	Cross-sectional	1761	755/1006	61.2 ± 9.3	Serum	Funduscopic examination	-	-	-	<0.6	1.00
											0.6–0.76	1.06 (0.69, 1.64)
											0.77–0.99	0.67 (0.42, 1.09)
											>0.99	0.55 (0.33, 0.91)
Kim, Eun Sook[29]	2014	Cross-sectional	1711	808/903	57.1 ± 10.5	Serum	n/r	-	-	-	Male:<0.70	1.00
											0.70–0.90	0.64 (0.50, 0.82)
											>0.90	0.45 (0.34, 0.60)
											Female:<0.60	1.00
											0.60–0.70	0.49 (0.37, 0.63)
											>0.70	0.59 (0.46, 0.76)
Xiaozhong, Yu[14]	2013	Case-control	373	190/183	59.6 ± 10.0	Serum	Funduscopic examination	0.44 ± 0.31	0.63 ± 0.28	-	-	-
Jie, Lai[30]	2013	Case-control	495	249/246	62.9 ± 10.7	Serum	Funduscopic examination	0.76 ± 0.26	0.82 ± 0.29	-	-	-
Chan, K. H[31]	2013	Cross-sectional	9795	6045/3750	62.0 ± 7.0	Plasma	n/r	-	-	-	Male: ≤0.35	1.00
											0.41–0.47	0.87 (0.68, 1.12)
											0.53–0.58	0.80 (0.62, 1.03)
											0.64–0.70	0.81 (0.61, 1.06)
											≥0.76	0.90 (0.70, 1.15)
											Female: ≤0.35	1.00
											0.41–0.47	0.86 (0.64, 1.15)
											0.53–0.58	1.12 (0.81, 1.56)
											0.64–0.70	1.25 (0.82, 1.91)
											≥0.76	0.60 (0.36, 1.02)
Yajing, Luo[32]	2013	Cross-sectional	246	118/128	62.1 ± 11.1	Serum	Funduscopic examination	0.65 ± 0.19	0.76 ± 0.20	-	-	-
Yuan, He[33]	2012	Case-control	708	355/352	56.7 ± 11.4	-	Funduscopic examination	0.70 ± 0.35	0.76 ± 0.35	-	-	-
Wei, Feng[34]	2012	Case-control	471	234/237	59.7 ± 10.0	Serum	Funduscopic examination	0.82 ± 0.28	0.85 ± 0.30	-	-	-
Wei, Du[35]	2012	Case-control	96	52/44	62.7 ± 10.3	Serum	Funduscopic examination	0.48 ± 0.10	0.68 ± 0.14	-	-	-
Hui, Xu[36]	2011	Case-control	86	51/35	63.2 ± 5.8	Serum	Funduscopic examination	0.50 ± 0.17	0.68 ± 0.26	-	-	-
Yasuda, Miho[37]	2011	Cohort study	486	-	-	Serum	Funduscopic examination	-	-	-	<0.60	1.00
											0.60–0.69	1.41 (0.56, 3.54)
											0.70–0.89	1.12 (0.41, 3.01)
											≥0.90	0.39(0.12, 1.30)
Cho, Ho Chan[13]	2011	Cross-sectional	102	54/48	59.6 ± 11.4	Serum	Funduscopic examination	0.59 ± 0.24	0.88 ± 0.47	0.17 (0.00, 1.00)^#^	-	-
Zhiyan, Su[39]	2010	Case-control	664	327/337	59.7 ± 9.73	Serum	Funduscopic examination	0.74 ± 0.27	0.90 ± 0.33	0.20 (0.10, 0.37)^#^	-	-
Yumei, Jia[38]	2010	Case-control	1062	571/491	58.2 ± 12.2	Serum	Funduscopic examination	0.60 ± 0.22	0.78 ± 0.23	0.21 (0.10, 0.46)^#^	-	-
Huang, E. J[18]	2006	Case-control	257	130/127	60.0±11.0	Serum	Funduscopic examination	0.73 ± 0.33	0.69 ± 0.37	-	-	-

^a^, As a continuous variable, the effect of bilirubin on DR;

^b^, As a categorical variable, the effect of bilirubin on DR;

TBL: total bilirubin level; n/r = not reported;

^#^, The OR was adjusted.

### Quality analysis

We assessed the quality of the included studies using seven items, which were selected from the STROBE statement [20]. Twenty-one studies were assessed as high quality and three studies were assessed as moderate quality. The detailed results on quality assessment of the included studies are shown in Table 2.

**Table 2:** The quality analysis of the included studies in the meta-analysis.

Study	Objectives	Study design	Setting	Participants	Variables	Statistical methods	Main results
Dan, Zhang(2015)[23]	●	●	●	●	●	●	●
Wei, Wei(2015)[15]	●	●	●	●	●	●	●
Fang, Chen(2015)[24]	●	●	◎	●	●	●	●
Junwei, Cai(2015)[19]	●	●	●	●	●	●	●
Sekioka, Risa(2015)[16]	●	●	●	●	●	●	●
Hamamoto, S(2015)[25]	●	●	●	●	●	●	●
Dave, Apoorva(2015)[26]	●	●	●	●	●	●	●
Xiaojing, Shang(2014)[17]	●	●	●	●	●	●	●
Wei, Peng(2014)[27]	●	●	●	●	●	●	●
Najam, Syeda Sadia(2014)[28]	●	●	●	●	●	●	●
Kim, Eun Sook(2014)[29]	●	●	●	●	●	●	●
Xiaozhong, Yu(2013)[14]	●	●	●	●	●	●	●
Jie, Lai(2013)[30]	●	●	●	●	●	●	●
Chan, K. H(2013)[31]	●	●	●	●	●	●	◎
Yajing, Luo(2013)[32]	●	●	●	●	●	●	●
Yuan, He(2012)[33]	●	●	◎	●	●	●	●
Wei, Feng(2012)[34]	●	●	●	●	●	●	●
Wei, Du(2012)[35]	●	●	●	●	●	●	●
Hui, Xu(2011)[36]	●	●	●	●	●	●	●
Yasuda, Miho(2011)[37]	●	●	●	●	●	●	●
Cho, Ho Chan(2011)[13]	●	●	●	●	●	●	●
Zhiyan, Su(2010)[39]	●	●	●	●	●	●	●
Yumei, Jia(2010)[38]	●	●	●	●	●	●	●
Huang, E. J(2006)[18]	●	●	●	●	●	●	●

●, the item was described in detail;

◎, the item was described partly.

### Overall and subgroup analysis

**Fig 2:**
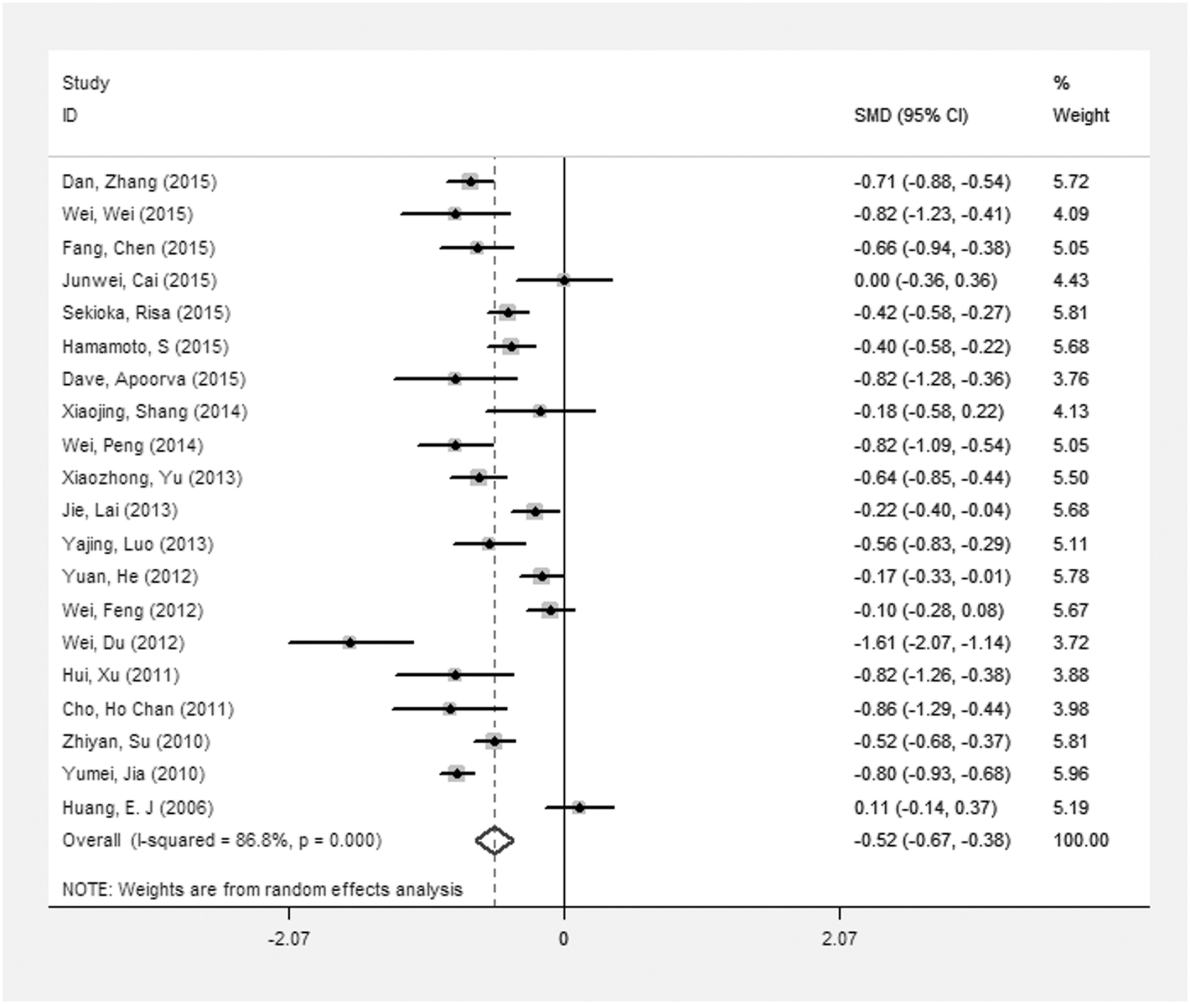
The SMD on the relationship between TBL and the risk of DR.

Twenty of 24 studies [13–19, 23–27, 30, 32–36, 38, 39] had calculated the pooled SMD, and the results showed that TBL in the DR group was lower than that in the NDR group (SMD: –0.52, 95% CI: –0.67, –0.38), see Fig 2. As there was obvious heterogeneity (I^2^ = 86.8%), we performed a subgroup analysis, and the results showed that TBL in the DR group was lower than that in the NDR group.

**Fig 3:**
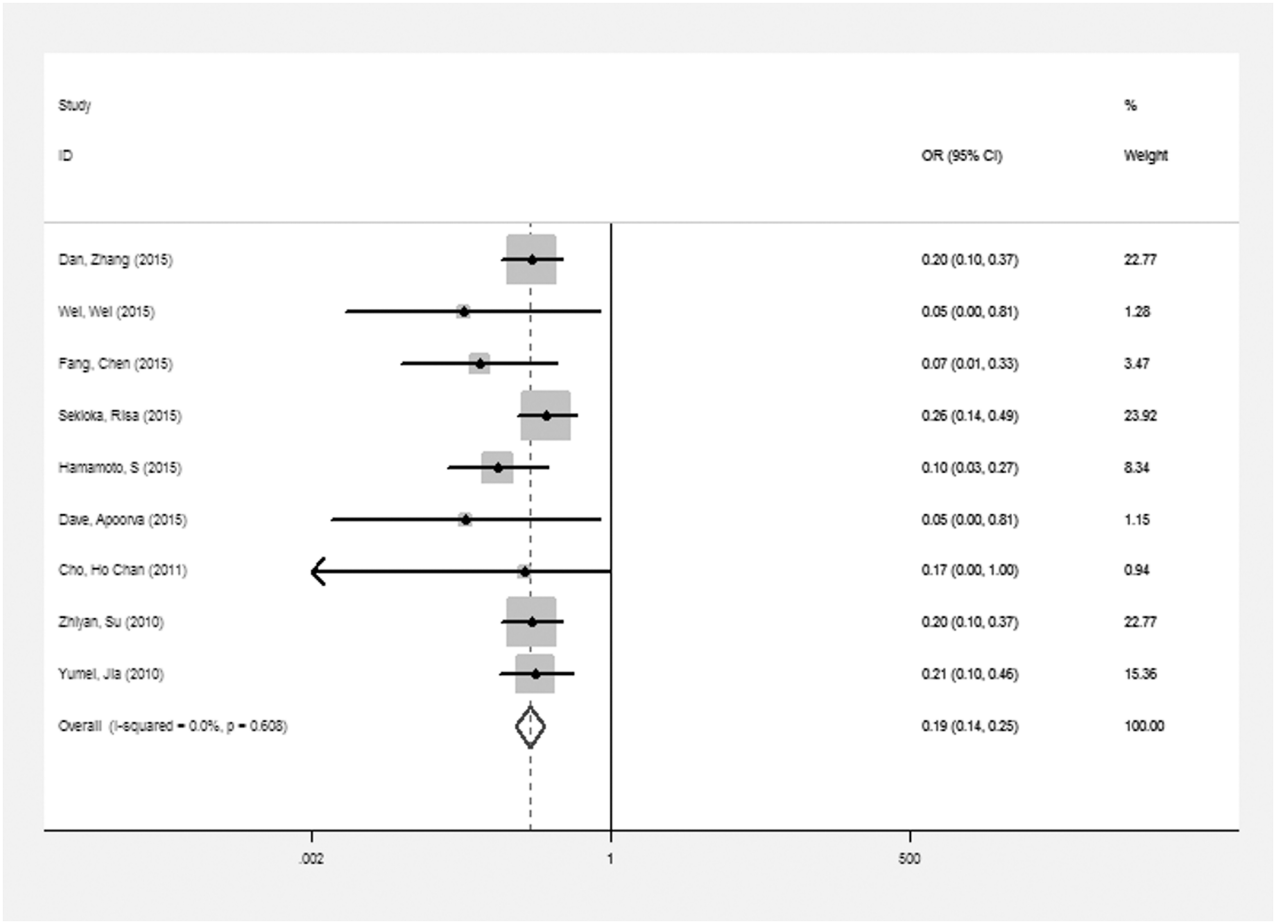
The pooled ORs on the relationship between TBL and the risk of DR.

Nine of 24 studies [13, 15, 16, 23–26, 38, 39] had calculated the pooled ORs, and the results showed that there was a significant negative relationship between TBL and the risk of DR (OR: 0.19, 95% CI: 0.14, 0.25), see Fig 3. We found that the ORs in 8 studies were adjusted, the OR in only one study [15] was not adjusted. The pooled adjusted OR also showed that there was a significant negative relationship between TBL and the risk of DR (OR: 0.16, 95% CI: 0.10, 0.22; I^2^ = 0). We also performed a subgroup analysis, and the results showed that there was a significant negative relationship between TBL and the risk of DR.

The detailed results of the overall and subgroup analyses on the relationship between TBL and the risk of DR are shown in Table 3.

**Table 3:** The SMD and pooled ORs on the relationship between TBL and the risk of DR.

	TBL in DR and NDR					TBL and risk of DR				
	No. of study	SMD (95%CI)	I^2^ (%)	P for heterogeneity	P for Egger’s test	No. of study	OR (95%CI)	I^2^ (%)	P for heterogeneity	P for Egger’s test
**Overall**	20	-0.52 (-0.67, -0.38)	86.8	0.000	0.547	9	0.19 (0.14,0.25)	0.0	0.608	0.017
**Year of publication**										
after 2013	12	-0.52 (-0.65, -0.38)	72.3	0.000		6	0.18 (0.12,0.26)	18.8	0.291	
before 2012	8	-0.56 (-0.85, -0.27)	93.3	0.000		3	0.20 (0.12,0.33)	0.0	0.982	
**Study design**										
case-control	16	-0.53 (-0.70, -0.35)	89.2	0.000		6	0.18 (0.12,0.26)	0.0	0.613	
cross-sectional	4	-0.46 (-0.56, -0.36)	35.7	0.198		3	0.20 (0.12,0.34)	24.9	0.264	
**Number of subjects**										
< 250	10	-0.70 (-0.94, -0.46)	76.5	0.000		5	0.20 (0.15,0.27)	0.0	0.614	
≥ 250	10	-0.39 (-0.57, -0.22)	90.4	0.000		4	0.07 (0.02,0.21)	0.0	0.934	
**Gender**										
male/female ≥ 1	13	-0.55 (-0.75, -0.36)	88.6	0.000		7	0.18 (0.12,0.26)	4.1	0.395	
male/female < 1	7	-0.49 (-0.70, -0.28)	84.5	0.000		2	0.20 (0.13,0.31)	0.0	1.000	
**Age**										
< 60	13	-0.54 (-0.71, -0.37)	85.9	0.000		7	0.18 (0.12,0.26)	0.0	0.735	
≥ 60	7	-0.50 (-0.76, -0.25)	88.0	0.000		2	0.20 (0.12,0.34)	62.2	0.104	
**Quality of study**										
high	18	-0.54 (-0.69, -0.39)	86.6	0.000		8	0.19 (0.14,0.26)	0.0	0.695	
moderate	2	-0.40 (-0.88, -0.08)	88.8	0.003		1	0.07 (0.01,0.33)	-	-	

TBL: total bilirubin level.

### Sensitivity analysis

We perform a sensitivity analysis to assess the stability and reliability of the results. After removing each single study from the pooled analysis, both the SMD and pooled ORs were unaffected (S1 and S2 Tables).

### Dose-response relationship between TBL and the risk of DR

**Fig 4:**
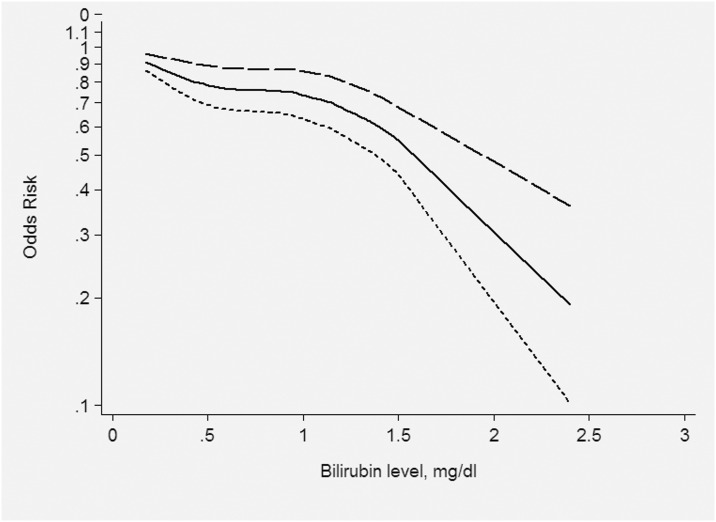
Dose—response analysis between TBL and the risk of DR.

Six of 24 [15, 26, 28, 29, 31, 37] included information on dose-response which reflected the relationship between TBL and the risk of DR. We found a nonlinear relationship between TBL and the risk of DR (P = 0.017), see Fig 4.

### Publication bias

As shown in Table 3, visual inspection of the Begg’s funnel plot or Egger’s test suggested no publication bias in SMD (S1 Fig), but publication bias was observed in the pooled ORs (S2 Fig). We performed the trim and fill method, and the results also showed a significant association between TBL and the risk of DR (OR: 1.76, 95% CI: 1.60–1.94).

## Discussion

DR is a common microvascular complication in T2DM patients. The pathogenesis of DR is not yet fully understood and there is no effective cure [40, 41]. Early detection and treatment can significantly delay the development of DR and preserve vision in most patients. The prevention of diabetic blindness has become an important public health problem worldwide [42]. In recent years, research on the relationship between bilirubin and DR has gradually increased, but the results are inconsistent. Therefore, we performed a meta-analysis to investigate the relationship between TBL and DR. This meta-analysis not only compared TBL between a DR and NDR group, but investigated the dose-response relationship between TBL and the risk of DR. Briefly, we found that TBL in the DR group was lower than that in the NDR group, and there was a significant negative relationship between TBL and the risk of DR. The dose-response trend suggested that increased TBL may delay DR progression.

Heterogeneity had an impact on the combined effect of the included studies. First, we used strict inclusion and exclusion criteria in the literature selection process. Second, we assessed the quality of included studies by selecting seven items from the STROBE statement, which provided a checklist of items on how to report observational research well. There were 21 high quality and three moderate quality studies. Third, because there were other potential confounding factors (year of publication, study design, number of subjects, gender, age), we carried out a subgroup analysis to determine the source of heterogeneity. In each subgroup analysis, we also found that TBL in the DR group was lower than that in the NDR group, and there was a significant negative relationship between TBL and the risk of DR. Fourth, we carried out a sensitivity analysis to assess the reliability of our meta-analysis. The results showed that no study influenced the combined effect. Therefore, our results were reliable.

Although all traditional risk factors (hyperglycemia, dyslipidosis, hypertension and duration of diabetes) are associated with the development and progression of DR [43], the change in serum redox status in T2DM patients is an additional risk factor. Research has found that endothelial dysfunction and hyperglycemia are associated with lipid peroxidation [44, 45]. To date, the underlying protective mechanism of bilirubin on DR is not yet fully understood, and a hypothesis has been proposed. Bilirubin is recognized as an important endogenous antioxidant, and has strong antioxidant and anti-inflammatory effects on the microvasculature [7, 13, 46]. A series of biochemical changes take place in the retinal microvasculature, including oxidative stress, the polyol pathway, protein kinase C activation, and advanced glycation end product formation, which are mainly due to long-term high blood glucose [47]. Oxidative stress and inflammation play important roles in the pathogenesis of DR [47, 48]. Biochemical reactions induced by oxidative stress change the function and structure of the retinal microvasculature. These functional changes include basement membrane thickening, microvascular cell loss, capillary closure, and cellular capillary formation. The structural changes include altered blood flow, loss of intercellular junctions, and increased vessel permeability [49]. The results of an animal study indicated that inhibition of inflammation may inhibit the progression of early stage DR [47]. Thus, high TBL could inhibit oxidative stress and inflammation processes and delay or interrupt development of DR.

To reduce the effects of the relevant factors on the results, we extracted and analyzed the adjusted OR to unadjusted OR. The ORs were adjusted to take into account common confounders (such as gender, age, BMI and biochemical indicators), this ensured the stability of the results of our meta-analysis. We found that the ORs in 8 studies were adjusted and the OR in only one study was not adjusted. We also carried out a subgroup analysis and the results were stable. Given the effect of liver disease on TBL, 22 of 24 studies excluded patients with liver disease, and only two studies did not clearly indicate whether patients with liver disease were excluded. These two studies were of high quality as evaluated by quality assessment, and we considered the results of these studies credible. During the sensitivity analysis, we excluded these two studies and the results were also stable. There are some limitations in our meta-analysis that should be taken into account. First, the combined effects were based on observational studies (SMD: 16 cross-sectional studies and four case-control studies; OR: six cross-sectional studiesand three case-control studies), and this may make the results unreliable. Therefore, we carried out a subgroup analysis and found that the combined effects in the cross-sectional and case—control studies were consistent with the overall results. Second, DR is a multi-factorial disease and influenced by both genetic and environmental risk factors, and most of the included studies were not adjusted by confounders, especially duration of diabetes. The risk of DR increased with longer duration of diabetes, and we were unable to extract the data from the included studies in our meta-analysis. Third, we found that only one cohort study analyzed the relationship between bilirubin level and the risk of DR, the other studies were cross-sectional and case-control studies. The cohort study did not find a significant association between bilirubin level and the risk of DR. Toyoshi Inoguchi et al [50] first reported a lower prevalence of DR in patients with diabetes and Gilbert syndrome, but this was also a cross-sectional study. Therefore, long-term cohort studies with large populations are needed to confirm the relationship between bilirubin level and the risk of DR.

In summary, the results of our meta-analysis indicate that higher TBL may be protective against DR in subjects with diabetes, and TBL could be used as a biomarker to predict the risk of DR. In addition, due to the limitations in this meta-analysis, long-term, large-scale studies are needed to confirm our results.

## Supporting Information

S1 Fig The funnel plot of the 20 studies on SMD. (TIF)

S2 Fig The funnel plot of the 20 studies on the pooled ORs. (TIF)

S1 PRISMA Checklist (DOC)

S1 Table Sensitivity analysis on SMD by removing each study in each model. (DOCX)

S2 Table Sensitivity analysis on the pooled ORs by removing each study in each model. (DOCX)
