# Supplementary figures and images for: The Negative Relationship between Bilirubin Level and Diabetic Retinopathy: A Meta-Analysis
Source: PLoS One. 2016 Aug 29;11(8):e0161649. doi: 10.1371/journal.pone.0161649 (PMC5003343; doi:10.1371/journal.pone.0161649)

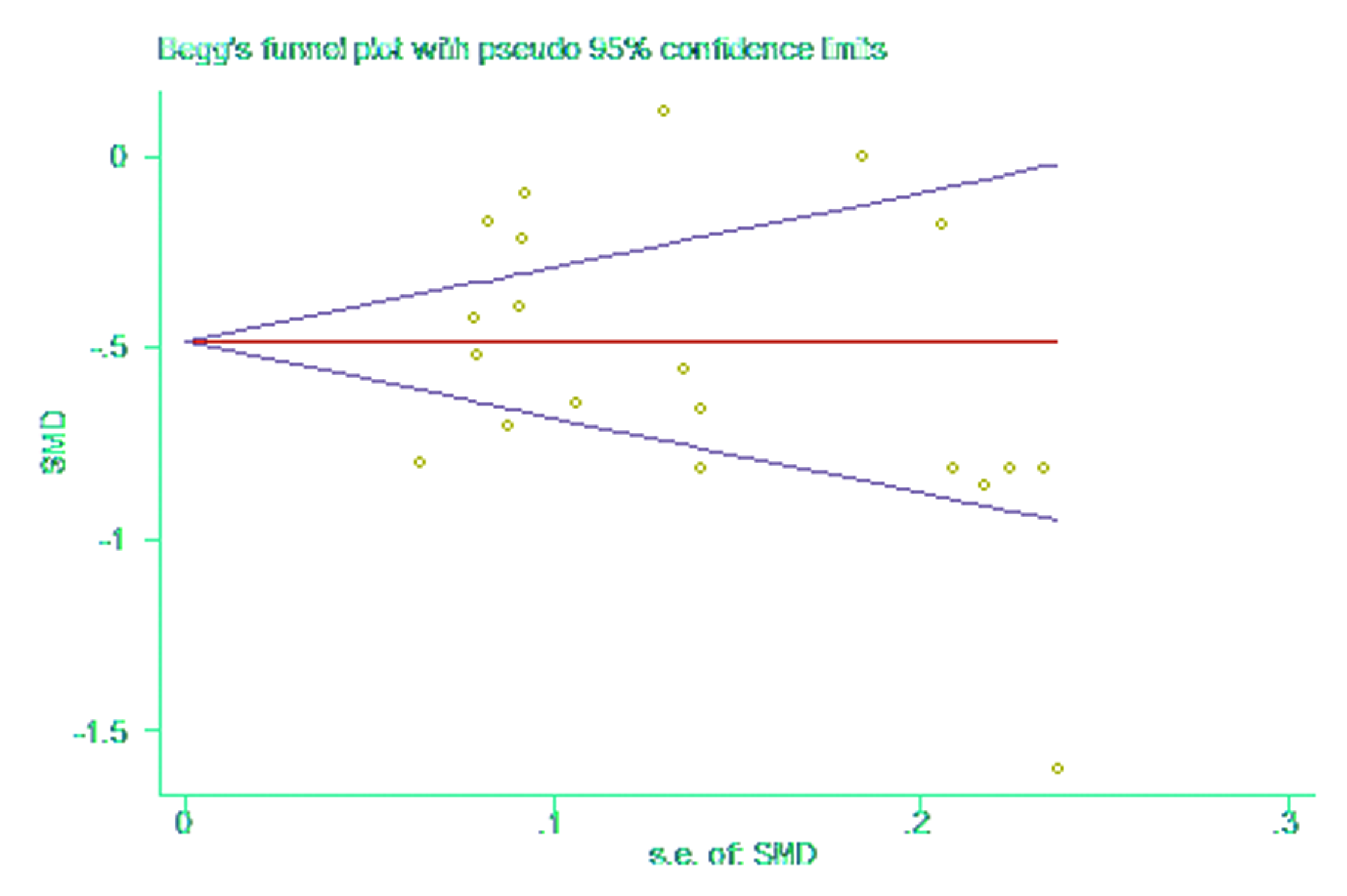

Supplement: S1 Fig — (TIF) [file pone.0161649.s001.tif]

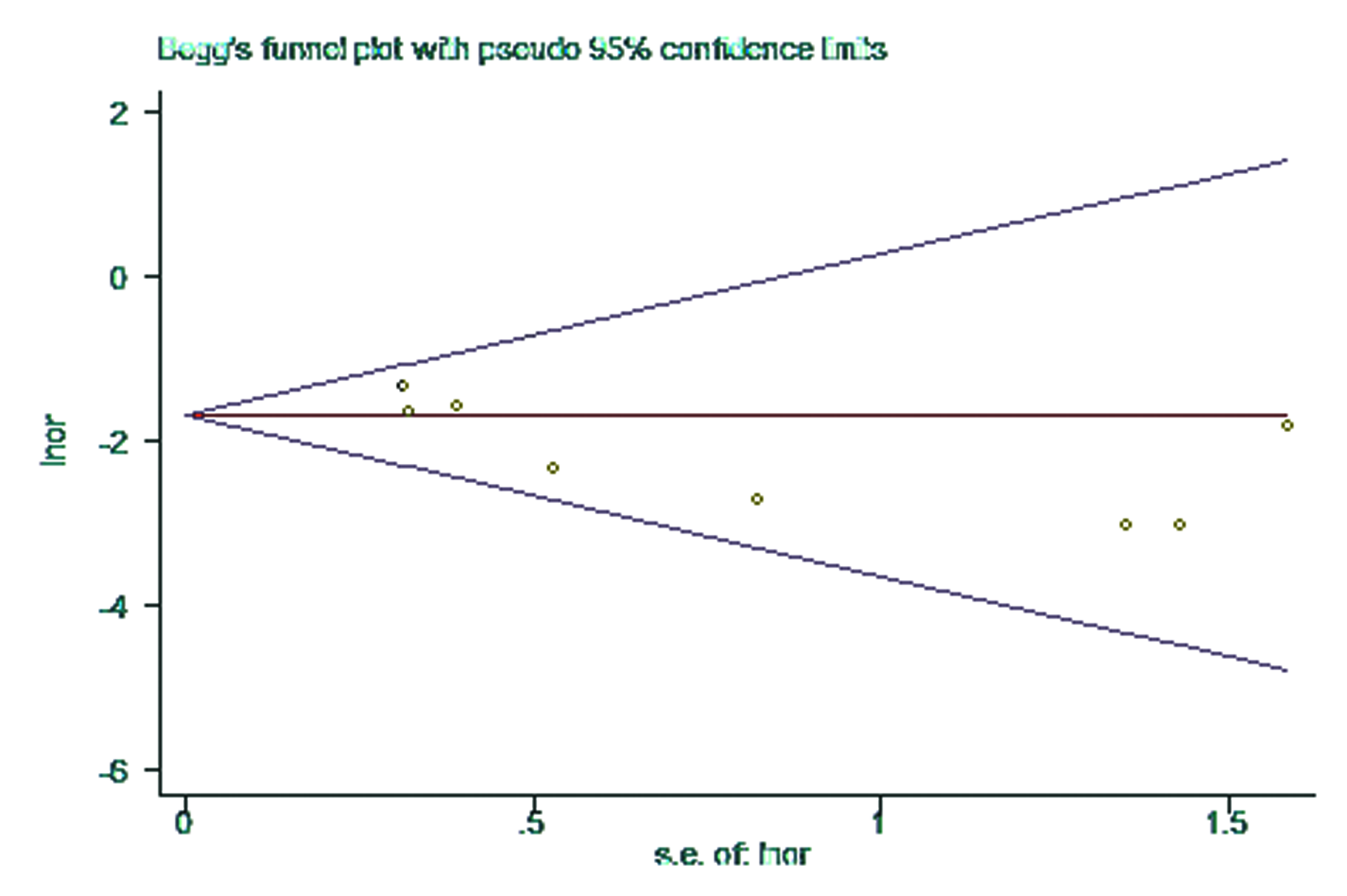

Supplement: S2 Fig — (TIF) [file pone.0161649.s002.tif]
