# Supplementary material for: The Negative Relationship between Bilirubin Level and Diabetic Retinopathy: A Meta-Analysis
Source: PLoS One. 2016 Aug 29;11(8):e0161649. doi: 10.1371/journal.pone.0161649 (PMC5003343; doi:10.1371/journal.pone.0161649)
Supplement: S1 Table — (DOCX) [file pone.0161649.s004.docx]

**S1 Table. Sensitivity analysis on SMD by removing each study in each model.**

| **Study omitted** | **SMD** | **95% CI** | |
| --- | --- | --- | --- |
|  |  | **Lower CI Limit** | **Upper CI Limit** |
| Dan, Zhang (2015) [23] | -0.51 | -0.66 | -0.37 |
| Wei, Wei (2015) [15] | -0.51 | -0.66 | -0.37 |
| Fang, Chen (2015) [24] | -0.52 | -0.66 | -0.37 |
| Junwei, Cai (2015) [19] | -0.55 | -0.69 | -0.41 |
| Sekioka, Risa (2015) [16] | -0.53 | -0.68 | -0.38 |
| Hamamoto, S (2015) [25] | -0.53 | -0.68 | -0.38 |
| Dave, Apoorva (2015) [26] | -0.51 | -0.66 | -0.37 |
| Xiaojing, Shang (2014) [17] | -0.54 | -0.68 | -0.40 |
| Wei, Peng (2014) [27] | -0.51 | -0.65 | -0.36 |
| Xiaozhong, Yu (2013) [14] | -0.52 | -0.67 | -0.37 |
| Jie, Lai (2013) [30] | -0.54 | -0.69 | -0.40 |
| Yajing, Luo (2013) [32] | -0.52 | -0.67 | -0.38 |
| Yuan, He (2012) [33] | -0.55 | -0.69 | -0.40 |
| Wei, Feng (2012) [34] | -0.55 | -0.69 | -0.41 |
| Wei, Du (2012) [35] | -0.48 | -0.61 | -0.35 |
| Hui, Xu (2011) [36] | -0.51 | -0.66 | -0.37 |
| Cho, Ho Chan (2011) [13] | -0.51 | -0.65 | -0.37 |
| Zhiyan, Su (2010) [39] | -0.53 | -0.68 | -0.37 |
| Yumei, Jia (2010) [38] | -0.51 | -0.65 | -0.36 |
| Huang, E. J (2006) [18] | -0.56 | -0.69 | -0.42 |
| Overall | -0.52 | -0.67 | -0.38 |
