# Supplementary material for: The Negative Relationship between Bilirubin Level and Diabetic Retinopathy: A Meta-Analysis
Source: PLoS One. 2016 Aug 29;11(8):e0161649. doi: 10.1371/journal.pone.0161649 (PMC5003343; doi:10.1371/journal.pone.0161649)
Supplement: S2 Table — (DOCX) [file pone.0161649.s005.docx]

**S2 Table. Sensitivity analysis on the pooled ORs by removing each study in each model.**

| **Study omitted** | **OR** | **95% CI** | |
| --- | --- | --- | --- |
|  |  | **Lower CI Limit** | **Upper CI Limit** |
| Dan, Zhang (2015) [23] | 0.18 | 0.13 | 0.26 |
| Wei, Wei (2015) [15] | 0.19 | 0.14 | 0.26 |
| Fang, Chen (2015) [24] | 0.19 | 0.14 | 0.26 |
| Sekioka, Risa (2015) [16] | 0.17 | 0.12 | 0.24 |
| Hamamoto, S (2015) [25] | 0.20 | 0.14 | 0.27 |
| Dave, Apoorva (2015) [26] | 0.19 | 0.14 | 0.26 |
| Cho, Ho Chan (2011) [13] | 0.19 | 0.14 | 0.25 |
| Zhiyan, Su (2010) [39] | 0.18 | 0.13 | 0.26 |
| Yumei, Jia (2010) [38] | 0.18 | 0.13 | 0.25 |
| Overall | 0.19 | 0.14 | 0.25 |
